# Identification and expression analysis of the lipid phosphate phosphatases gene family reveal their involvement in abiotic stress response in kiwifruit

**DOI:** 10.3389/fpls.2022.942937

**Published:** 2022-08-24

**Authors:** Yaming Yang, Lijuan Chen, Gen Su, Fangfang Liu, Qiang Zeng, Rui Li, Guili Cha, Cuihua Liu, Libo Xing, Xiaolin Ren, Yuduan Ding

**Affiliations:** ^1^College of Horticulture, Northwest Agricultural and Forestry University, Yangling, China; ^2^Institute of Horticulture, Sichuan Academy of Agricultural Sciences, Chengdu, China

**Keywords:** abiotic stress, phytohormone, lipid phosphate phosphatases, gene expression, kiwifruit (*Actinidea deliciosa*)

## Abstract

Lipid phosphate phosphatases (LPPs) are a key enzyme in the production and degradation of phosphatidic acid (PA), which plays an important role in plant growth, development, stress resistance and plant hormone response. Thus far, little is known about the LPP family genes in kiwifruit (*Actinidia* spp.). According to this study, 7 members in the *AcLPP* family were identified from the whole genome of kiwifruit, the subcellular localization predictions were mainly on the plasma membrane. Chromosomal localization analysis showed that the *AcLPP* genes were unevenly distributed on 5 chromosomes, it was determined to have undergone strong purifying selection pressure. There were 5 duplicate gene pairs and all underwent segmental duplication events. The LPP genes of kiwifruit were conserved when compared with other plants, especially in terms of evolutionary relationships, conserved motifs, protein sequences, and gene structures. *Cis*-regulatory elements mainly included hormone response elements and abiotic response elements. Functional annotation of GO revealed that *AcLPP* genes were closely related to phosphatase/hydrolase activity, phosphorus metabolism and dephosphorylation. *AcLPP* genes family were predicted to be targets of miRNA. Transcript level analysis revealed that the *AcLPP* family played diverse functions in different tissues and during growth, development, and postharvest storage stages. qPCR analysis showed that the members of *AcLPP* gene family might be regulated by ETH, ABA, GA_3_, and IAA hormone signals. The family members were regulated by the stress of salt stress, osmotic stress, cold stress, and heat stress. These results would provide a basis and reference for studying the agricultural characteristics of kiwifruit and improving its stress resistance.

## Introduction

Phospholipids widely exist in organisms (plants and animals), they are an important part of the cell membrane and the main source of oil crops. Lipids are also distributed in fruits and seeds of plants, they participate in signal transduction, membrane biogenesis, energy storage, and other biological processes (Su et al., 2022). Triacylglycerols (TAGs) are plant oil stores of the primary modalities.

Lipid phosphate phosphatase (LPP) catalyzes the generation of Phosphatidic acids (PAs) from diacylglycerol pyrophosphate (DGPP) and further phosphorylates PA to form diacylglycerol (DAG) (Carman and Han, 2006). The phosphatidic acid phosphatases (PAPs) are important dephosphorylation enzymes that dephosphorylate the substrate of PAs. The PAPs can be divided into two types: the PAP1 and PAP2 (also known as lipid phosphate phosphatase, LPP). Research has shown that PAP1 and LPP belong to different families, which are very different in molecular structure, biochemical properties, and the mechanisms of their regulation. The PAP1 are soluble enzymes with high specificity for the substrates, their reactions always depend on Mg^2+^ and can be inhibited by N-ethylmaleimide (NEM). Research shows the LPPs can hydrolyze a series of lipid phosphate substances containing single ester bonds and are located on the plasma membrane. the LPP reactions do not depend on Mg^2+^ and are usually insensitive to NEM (Carman, 2019). The LPP is a key enzyme that catalyzes the degradation and production of PAs. The PAs participate in the biological and metabolism of lipids and are the precursors of phospholipids in lipid synthesis and metabolism (Arisz et al., 2009; Munnik and Testerink, 2009) and are involved in fruit ripening, leaf senescence, seed germination, and response to various biotic and abiotic stresses (Hong et al., 2010; Paradis et al., 2011; Abu Sadat et al., 2014).

The plant LPP enzymes participate in lipid metabolism and regulate plant growth, development, and stress response. A study has shown that PA is involved in negative regulation of the ethylene signaling pathway and able to promote ethylene production, which acts by binding to the kinase domain of constitutive triple reaction 1 (CTR1) (Testerink et al., 2007). For example, in cowpea, *VuPAPa* and *VuPAPb* might be involved in the modification of cell membrane lipids in response to drought stress (Franca et al., 2008). In *Arabidopsis thaliana, AtLPP2* induced abscisic acid (ABA) signal transduction and stomatal movement, resulting in decreased drought resistance in *Arabidopsis thaliana*. *AtLPP2* also played a negative regulatory role in seed germination inhibition induced by ABA (Katagiri et al., 2005; Paradis et al., 2011). Physiological analysis shows that the accumulation of PA triggered the early signal transduction, leading to the ABA response during seed germination and regulated stomatal movement (Katagiri et al., 2005). LPP was also involved in the reaction process of plant pathogens. The *MoLPP3* and *MoLPP5* could significantly affect the invasion and growth of rice blast in rice (Abu Sadat et al., 2014; Carman, 2019). In another study, *AtLPP1* was mainly expressed in leaves and roots and was induced by stress responses such as ultraviolet light and hypersensitive pathogenic proteins. However, *AtLPP2* was expressed in all tissues (Wu et al., 1996). *AtLPP4* was significantly upregulated in pollens, implying that it might be involved in plant pollen development (Pleskot et al., 2012). In rapeseed, the expression levels of *BnLPP2A* and *BnLPP2B* were higher in stigmata. *BnLPP4A* expression was higher in stamens. *BnLPP4B* was expressed at higher levels in roots and *BnLPP3C* was expressed at higher levels in stems (Su et al., 2021). LPPs are lipsoluble proteins found mainly in the plasma membrane, and the VuPAPa protein had been proved to be located in the chloroplast *in vitro* (Franca et al., 2008). In Arabidopsis, AtLPP2 had been localized in the cell membrane (Katagiri et al., 2005).

The kiwifruit (*Actinidia* spp.) is rich in vitamins, minerals, and other nutrients, and is also beneficial to human health and profitable in agricultural production (Huang and Ferguson, 2001). There are few studies on LPPs in pomology and they are rarely about kiwifruit. Therefore, it is interesting to explore the function of the LPP family in kiwifruit growth, development, stress response, fruit ripening and senescence. In this study, the LPP genes family of kiwifruit was analyzed at the whole genome and the phylogenetic relationship, homologous relationship, gene structure, *cis*-acting elements, miRNA regulator prediction, regulatory network, and evolutionary relationship were analyzed. In addition, the transcriptional level of *AcLPP* under hormonal treatment and abiotic stress were detected by qPCR analysis. The results could provide a foundation for further study on the function of the AcLPP gene family.

## Materials and methods

### Processing of kiwifruit RNA-seq data

The original transcriptome sequencing data (raw reads data) were obtained from NCBI's SRA database, They were samples from fruit tissues of development period, postharvest storage period and different treatment conditions (PRJNA564374, CRA003106, PRJNA625794, and PRJNA638129). The original data was obtained by Trimmomatic (Bolger et al., 2014). Quality filtering was carried out to remove low-quality reads. Then, clean reads were aligned to a reference genome of *Actinidia chinensis* ‘Red5' (http://kiwifruitgenome.org/) by TopHat v2.0.9 (Trapnell et al., 2009), the gene expression FPKM value (fragments per kilobase of transcript per million mapped reads) was calculated by comparing the specific reads using Cuffdiff (Trapnell et al., 2012).

### Identification of members of LPP gene family in kiwifruit

To identify the member of the LPP gene family in kiwifruit, the 4 LPP amino acid sequences of *Arabidopsis thaliana* [AtLPP1 (*At2g01180*), AtLPP2 (*At1g15080*), AtLPP3 (*At3g02600*), and AtLPP4 (*At3g18220*)] were used as the query sequence to a BlastP search of kiwifruit ‘Red5' proteins sequence (parameter *E* <1e-5) (Pierrugues et al., 2001). In addition, the PAP2 domain (PF01569) of LPP was downloaded from the Pfam database (El-Gebali et al., 2019) using HMMER 3.0 software, and the PAP2 domain of candidate genes was further proved by Pfam database. Finally, 7 members in the *AcLPP* gene family were identified from the kiwifruit genome.

Information on sequence length, molecular weight, isoelectric point and predicted subcellular location was obtained from ExPasy website (http://web.expasy.org/protparam/; Gasteiger, 2005). The gene structure was analyzed by online website Gene Structure Display Server (GSDS: http://gsds.cbi.pku.edu.cn; Anyuan et al., 2007). Subcellular localization of the LPP protein was predicted online by Wolf PSORT (https://wolfpsort.hgc.jp; Paul et al., 2007).

### Bioinformatics analysis of members of LPP gene family in kiwifruit

The distribution of the conserved sequence motif of AcLPP family proteins in kiwifruit was analyzed by MEME online website (Bailey et al., 2009). The ClustalW was used to align the members of AcLPP family, and the adjacency method (neighbor-joining, NJ) of MEGA7.0 software was used to construct the phylogenetic tree, adopting the following parameters: the check parameter Bootstrap is repeated 1,000 times, and the mode is Poisson model (Larkin et al., 2007; Sudhir et al., 2016). To predict the *cis*-acting element, the 1,500 bp sequence of the upstream sequences at the initiation codon (ATG) of the AcLPPs genes family was truncated from the kiwifruit genome and submitted to the PlantCARE database (http://bioinformatics.psb.ugent.be/webtools/plantcare/html/) and PlantPAN 2.0 (http://plantpan2.itps.ncku.edu.tw/; Lescot et al., 2002; Chi-Nga et al., 2016). All *AcLPP* genes were mapped and illustrated in ‘Red5' kiwifruit chromosomes by Circos. The gene replication events were analyzed by MCScanX. The orthologous relationship LPPs genes of kiwifruit and other selected species was constructed by using Dual Synteny Plotter software. On the other hand, the paralogs relationship among *AcLPPs* genes of kiwifruit was analyzed, and the homologous map was constructed by Dual Synteny Plotter software (Chen et al., 2020). KaKs Calculator 2.0 software was used to estimate the values of non-synonyms (Ka), synonyms (Ks), and Ka/Ks ratio.

### Putative miRNA target prediction, protein-protein interaction network, and functional annotation analysis of AcLPP gene

In this study, the CDS sequences of AcLPP were submitted to PmiREN (https://www.pmiren.com/) to predict the target miRNA, and the E-value above 5.0 was selected, the score range was between 10 and 20, and other parameters were defaulted. The linkages between the predicted miRNAs and their corresponding target genes were demonstrated by Cytoscape software. AcLPP proteins were annotated by homology alignment using the eggnog website (http://eggnog-mapper.embl.de/), with evaluation 1e^−5^ as the other default. To predict the interaction between AcLPP protein and other related proteins, the AcLPP protein sequence was submitted to the String database. The reference species was set to kiwifruit, the confidence interval was 0.9, the first-level connection point was 20, and the other settings used were defaults. Cytoscape software was used to visualize the PPI network.

### Plant materials and stress treatment

To evaluate stress and hormone effects, the kiwifruit seedlings of *Actinidia chinensis* var. ‘Xuxiang' were planted in the greenhouse. The kiwifruit seedlings of 'Xuxiang' were treated with 4 different hormones. The kiwifruit seedlings (plant height of about 3 cm) were immersed in distilled water containing 20 μM ethephon (ETH), 20 μM gibberellin (GA_3_), 20 μ M Indoleacetic acid (IAA), and 20 μM abscisic acid (ABA) for 3 h, respectively. The kiwifruit seedlings in the control group were immersed in distilled water for 3 h, and the plant leaves were taken as materials and stored in liquid nitrogen. Abiotic stress treatment: using 2-month kiwifruit seed seedlings, under normal conditions, the growing plants were transferred and incubated at 4 ±1°C or 42 ±1°C for cold injury or heat stress. After 6 h of treatment, the samples were collected, and the untreated kiwifruit plants were used as the control. The plants were sprayed with 200 mM NaCl and 100 mM mannitol as salt stress and osmotic stress, respectively. After 6 h of treatment, the leaves were taken as the test material, and the untreated kiwifruit plants as the control.

Prime Script RT reagent Kit (Perfect Real Time) kit (Takara) was used to extract RNA, cDNA synthesis from leaves of kiwifruit under hormone stress and abiotic stress. Seven LPP genes of kiwifruit were analyzed by qRT-PCR with primers (Supplementary Table 1). Finally, gene expression levels were calculated by using the method of 2^−ΔΔCT^ (Livak and Schmittgen, 2002).

## Results

### Identification of members of AcLPP gene family members and their physicochemical properties

In the current study, a total of 7 genes in the *LPP* family containing complete *PAP2* functional domain were identified in kiwifruit genome. Based on the results of *Arabidopsis thaliana* Blastp and its chromosome position and the study of other species, they were named AcLPP1A ~ AcLPP4A, respectively (Table 1). The result showed that the 7 *AcLPP* genes were distributed on 5 different chromosomes: LG13, LG25, LG26, LG27, and LG29. The lengths of their open reading frame (ORF) were between 942 and 1,023 bp, the length of the proteins were 313–340 amino acids, respectively, and the molecular weight of protein was between 35.2 and 38.7 kD. Among them, *AcLPP1A* had a maximal AA molecular weight of 38.7 kD, and its ORF length was 1,023 bp. On the contrary, the *AcLPP4A* was minimally AA molecular weighted. Three members of the family (*AcLPP2A, AcLPP2B*, and *AcLPP3A*) had isoelectric points <7, which encode acidic amino acids, and the others were basic amino acids. The result of subcellular localization prediction showed that AcLPP was mainly distributed in the plasma membrane, otherwise, AcLPP2A was distributed in the endoplasmic reticulum and AcLPP4A in the cytoskeleton.

**Table 1:** The characteristics of the 7 AcLPP family members in kiwifruit.

**Gene name**	**Gene Id**	**Genomic position (bp)**	**CDS length (bp)**	**Protein length (aa)**	**MW**	**pI**	**Predicted Pfam domain**	**Subcellular location**
AcLPP1A	Acc14050	LG13: 902,467–902,467+	1,023	340	38.7	7.31	PAP2	Plas
AcLPP1B	Acc28469	LG25: 8,849,713–8,854,342-	993	330	37.6	7.3	PAP2	Plas
AcLPP1C	Acc31339	LG27: 3,864,494–3,868,437+	984	327	37.2	8.81	PAP2	Plas
AcLPP2A	Acc28468	LG25: 8,840,058–8,846,011-	969	322	36.6	6.55	PAP2	ER
AcLPP2B	Acc32927	LG29: 7,276,731–7,281,987+	966	321	35.9	6.29	PAP2	Plas
AcLPP3A	Acc30867	LG27: 4,876,375–4,883,612+	960	319	36.1	6.95	PAP2	Plas
AcLPP4A	Acc29275	LG26: 1,533,425–1,552,843+	942	313	35.2	7.33	PAP2	Cyto

In the genomic position, the positive (+) and negative (–) sign indicates the existence of gene on the positive and negative strand of that specific markers, respectively. CDS, coding DNA sequences; bp, base pair; MW, molecular weight; pI, isoelectric points; Plas, plasma membrane; ER, endoplasmic reticulum; Chlo, chloroplast; Cyto, cytoskeleton.

### Phylogenetic analysis and classification of AcLPP genes

To study the phylogenetic relationship of AcLPP proteins, multiple sequence alignments of 32 amino acid LPP domains from 5 species were carried out. Phylogenetic tree analysis of LPP gene family in kiwifruit and *Arabidopsis* showed that AcLPP genes in kiwifruit could be divided into four groups (groupI, groupII, groupIII, and groupIV) based on the sequence identity of amino acids (Figure 2B). The result showed that group I contained 10 LPP members: 3 *Actinidia Chinese* (Ac), 3 *Brassia napus* (Bn), 1 *B. rapa* (Bra), 2 *B. oleracea* (Bo), and 1 *Arabidopsis thaliana* (Ath), group II contained 7 LPP members (3 Ac, 2 Bn, 1 Bra, 1 Bo, and 1 At), group III contained 8 LPP members (1 Ac, 4 Bn, 2 Bra, and 1 At), and group IV contained 7 LPP members (1 Ac, 2 Bn, 2 Bra, 1 Bo, and 1 At). According to the phylogeny and historical relationship of species, the phylogenetic relationships of different species could be found. In the process of evolution, the phylogenetic tree divided AcLPP proteins into four branches, and the similar LPPs may have similar functions.

### Structure, conserved motif, and collinear analysis of AcLPP genes

**Figure 1:**
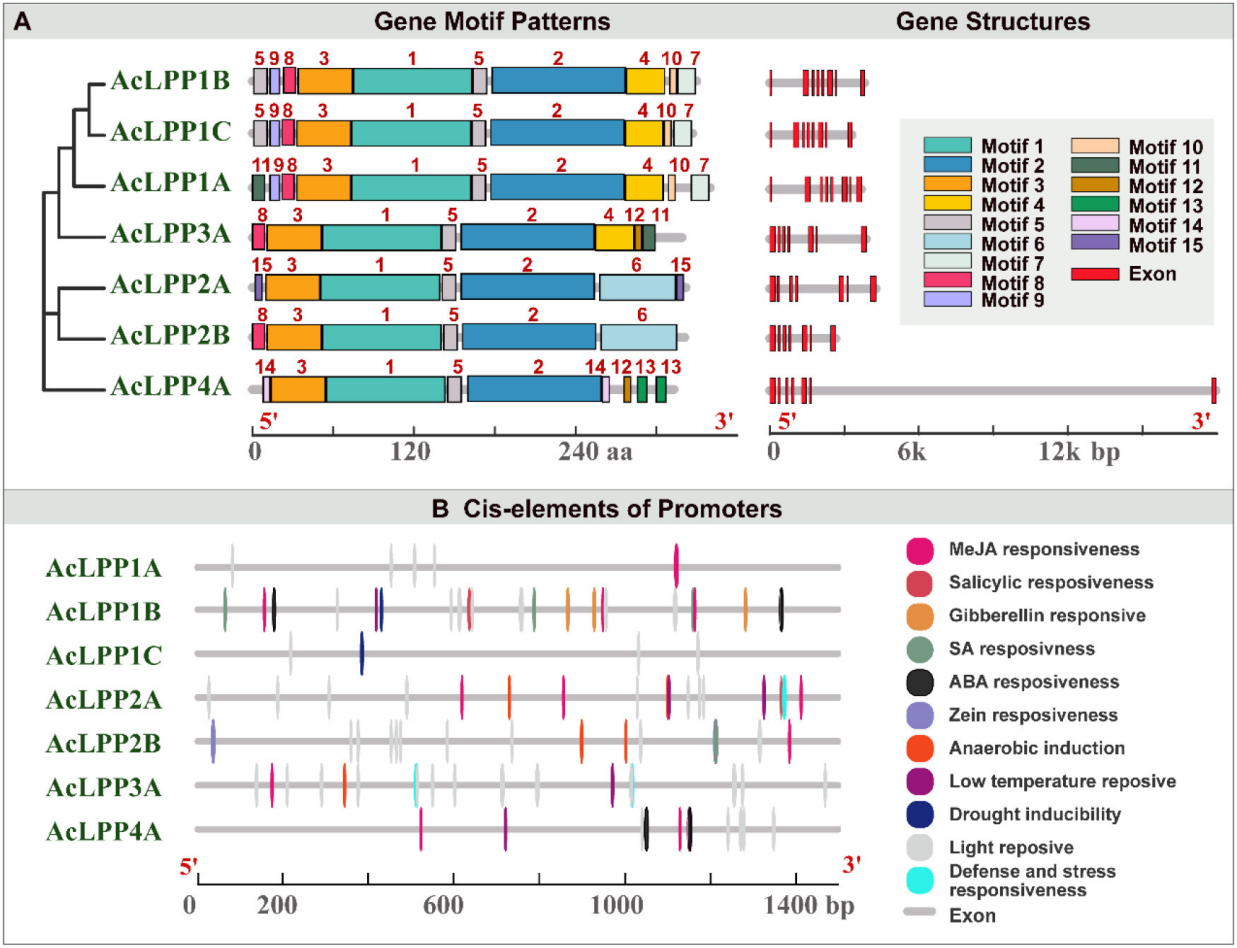
**(A)** The phylogenetic relationship, the gene structure and the conserved protein motif architecture of the 7 LPP family members in kiwifruit. The phylogenetic tree was constructed based on the full-length sequencing of kiwifruit LPP proteins using MEGA7.0 software. The motifs, numbered 1–15, displayed in different colored boxes. The sequence information for each motif is provided in Supplementary Table 2. The length of the protein could be estimated using the scale at the bottom. Exon–intron structure of kiwifruit LPP genes. Red boxes indicate UTR regions blackish-gray lines indicate intron. **(B)** The *cis*-elements related to different stress and hormone responses in the putative promoters of AcLPPs. The *cis*-elements with similar functions were displayed in the same color. Different color boxes show different identified *cis*-elements.

The analysis of exon-intron structure could provide important insights for the evolution of gene families. An adjacent phylogenetic tree was constructed to explore the exon-intron distribution pattern and its relation to the phylogenetic classification. As shown in Figure 1A, 7 AcLPP genes were divided into four subgroups according to the protein sequence differences of family members. Gene structure analysis showed that the AcLPP genes of the same subfamily had similar exon-intron distribution patterns (Figure 1A and Supplementary Table 2). The exon distribution of members of the *AcLPP* gene family had little difference, all of them had exons 7–8. These results provided important evidence for the reliability of gene structure analysis.

**Figure 2:**
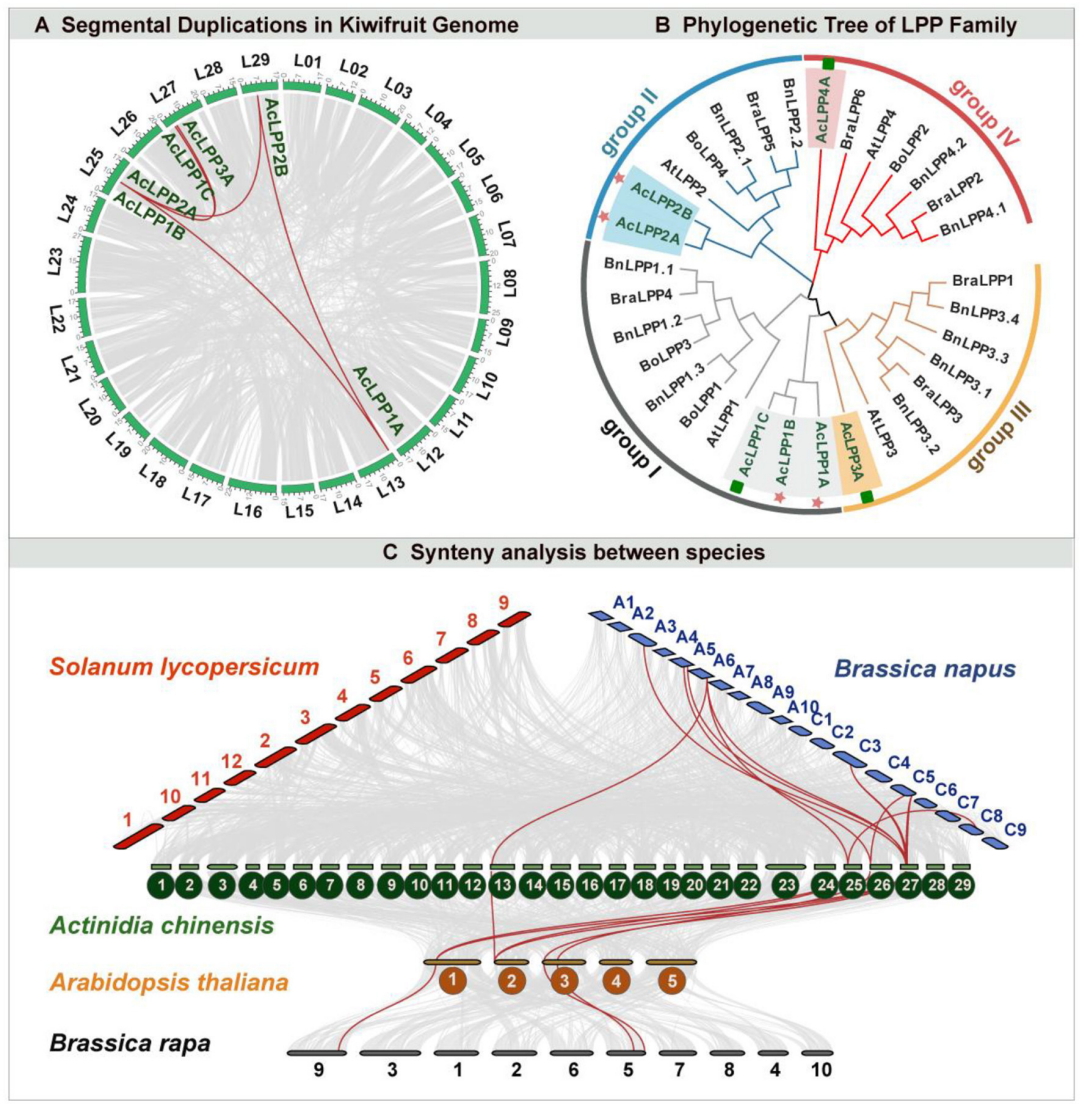
**(A)** Schematic representations of segmental duplications of AcLPP genes. Gray lines indicate all synteny blocks in kiwifruit genome between each chromosome, and the thick red lines indicate duplicated LPP gene pairs. The chromosome number is indicated at the bottom of each chromosome. Scale bar marked on the chromosome indicating chromosome lengths (Mb). **(B)** The phylogenetic tree of LPP gene family in *Brassia napus* (Bn), *B. oleracea* (Bo), *B. rapa* (Bra), *Arabidopsis thaliana* (Ath), and *Actinidia Chinese* (Ac). Different species LPP genes were clustered into four groups (Group I-IV) based on high bootstrap values signified with different background colors. The red star and green rectangle indicate that these genes belong to the subgenome, respectively. **(C)** Synteny analysis of AcLPP between other species. Syntenic genes of kiwifruit (*Actinidia chinensis*), Arabidopsis (*Arabidopsis thaliana*), *Brassica napus* (*B. napus*), tomato (*Solanum lycopersicum*), and *Brassica rapa* (*B. rapa*) are exhibited with red, Turquoise blue, and Cyan lines, respectively. Gray lines indicate the synteny blocks.

In addition, the conserved motif of AcLPP was analyzed by MEME online tool. Result (Figure 1A) showed that AcLPP members contained 15 conserved motifs. Three AcLPP proteins contain all 10 conserved motifs, which were mainly distributed in subgroup I, among which motifs 3, 1, 5, and 2 protein motifs were the most conserved and were contained in all family members. Due to the fluctuation of motif numbers between 6 and 10 and the change of conservative sequence position, there were some differences in protein motifs among members of different subfamilies. Motif domain analysis showed that the type and distribution of conserved motifs could be related to the diversity of gene function. The collinear relationship between kiwifruit AcLPP families was shown in Figure 2A. The result shows that there were 5 pairs of collinear relationships between kiwifruit LPP families (AcLPP1A & AcLPP2A, AcLPP1A & AcLPP2B, AcLPP1B & AcLPP1C, AcLPP2A & AcLPP2B, AcLPP2A & AcLPP3A). These 5 pairs of collinear relationships belonged to fragment replication of different chromosomes, and indicated that fragment replication exists between kiwifruit LPP genes. The results show that some LPP genes may be produced by fragment replication, and these replication events are the main derived force of LPP gene evolution. Ka and Ks values were used to evaluate the evolution rate of protein coding genes. The value of Ka/Ks ratio represented the type and evolution rate of gene selection pressure. If Ka/Ks = 1, the selection is neutral, Ka/Ks < 1 indicates purification selection, and Ka/Ks > 1 indicates positive selection (Supplementary Table 3). The Ka/Ks of kiwifruit LPP fragment replication gene pairs ranged from 0.091 to 0.280, indicating that AcLPP had a strong purifying selection pressure in the process of evolution.

To further analyze the gene replication relationship of AcLPP, comparative genomics analysis was carried out. The evolutionary relationships among kiwifruit, *Arabidopsis*, tomato, *Brassica rapa*, and *Brassica napus* were analyzed (Figure 2C). The result showed that the 5 LPP genes of kiwifruit (AcLPP1A, AcLPP2A, AcLPP3A, AcLPP4A, and AcLPP1C) had 7 pairs of LPP collinearity with *Arabidopsis*, 12 pairs of collinearity with *B. rapa* and no collinearity with tomato. In addition, there were 3 pairs of LPP collinearity between *B. rapa* and *Arabidopsis*. The result showed that the LPP gene family members of different species may come from the same ancestor and play a similar role in function. The number of AcLPP homologous genes between kiwifruit and *B. rapa* was more than that between tomato and *Arabidopsis*, which indicated that LPP had different homology among different species.

### Analysis of *cis*-acting elements of AcLPP gene

To further identify the *cis*-regulatory elements upstream of the AcLPP gene, the PlantCare tool was used to analyze the sequence of the 1,500 bp upstream of the translation initiation site of the AcLPP gene family. As shown in Figure 1B, at least 11 *cis*-regulatory elements were identified in the promoter of the AcLPPs genes. There are three main types of *cis*-regulatory factors, namely, hormone response elements, stress response, and light response related elements. There are six types of elements related to hormone response: abscisic acid response element, auxin response element, jasmonic acid response element, gibberellin response element and salicylic acid response element, and *cis*-acting elements related to drought stress, low temperature stress, defense, anaerobic induction, meristem expression, and light response (Supplementary Table 4). These hormone response elements and abiotic response elements indicate that AcLPP genes are widely involved in plant stress responses, thus improving the chances for organisms to better cope with adverse environmental conditions, highlighting the possible role of AcLPP genes in hormonal and stress response mechanisms.

### Functional annotation analysis of AcLPP genes

In order to understand the molecular function of AcLPP gene, the function of AcLPP protein can be described through the database established by EggNOG. Gene Ontology (GO) can be divided into three parts: molecular function (MF), biological process (BP), and cell composition (CC). GO annotation result revealed that the AcLPP family is involved in many biological functions (Supplementary Table 5). For instance, the GO-BP enrichment result revealed 19 enriched terms, including response to stimulus (GO:00508960), phospholipid metabolic process (GO:0006644), response to abiotic stimulus (GO:0009628), and so on. The GO-CC enrichment result displayed 12 enriched terms such as plasma membrane (GO:0005886), integral component of plasma membrane (GO:0005887); obsolete membrane part (GO:0044425), and so on. The GO-MF enrichment exposed eight enriched terms, including phosphatidate phosphatase activity (GO:0008195), hydrolase activity (GO:0016787), acid phosphatase activity (GO:0003993), and so on (Supplementary Table 5). The GO enrichment result verify that the AcLPP gene responds to phospholipid metabolic, abiotic stimulus, phosphatidate phosphatase activity, and hydrolase activity membrane part. The cell component is mainly in the plasma membrane, which is also consistent with the prediction result of subcellular localization.

### Genome-wide analysis interaction networks analysis of AcLPP

**Figure 3:**
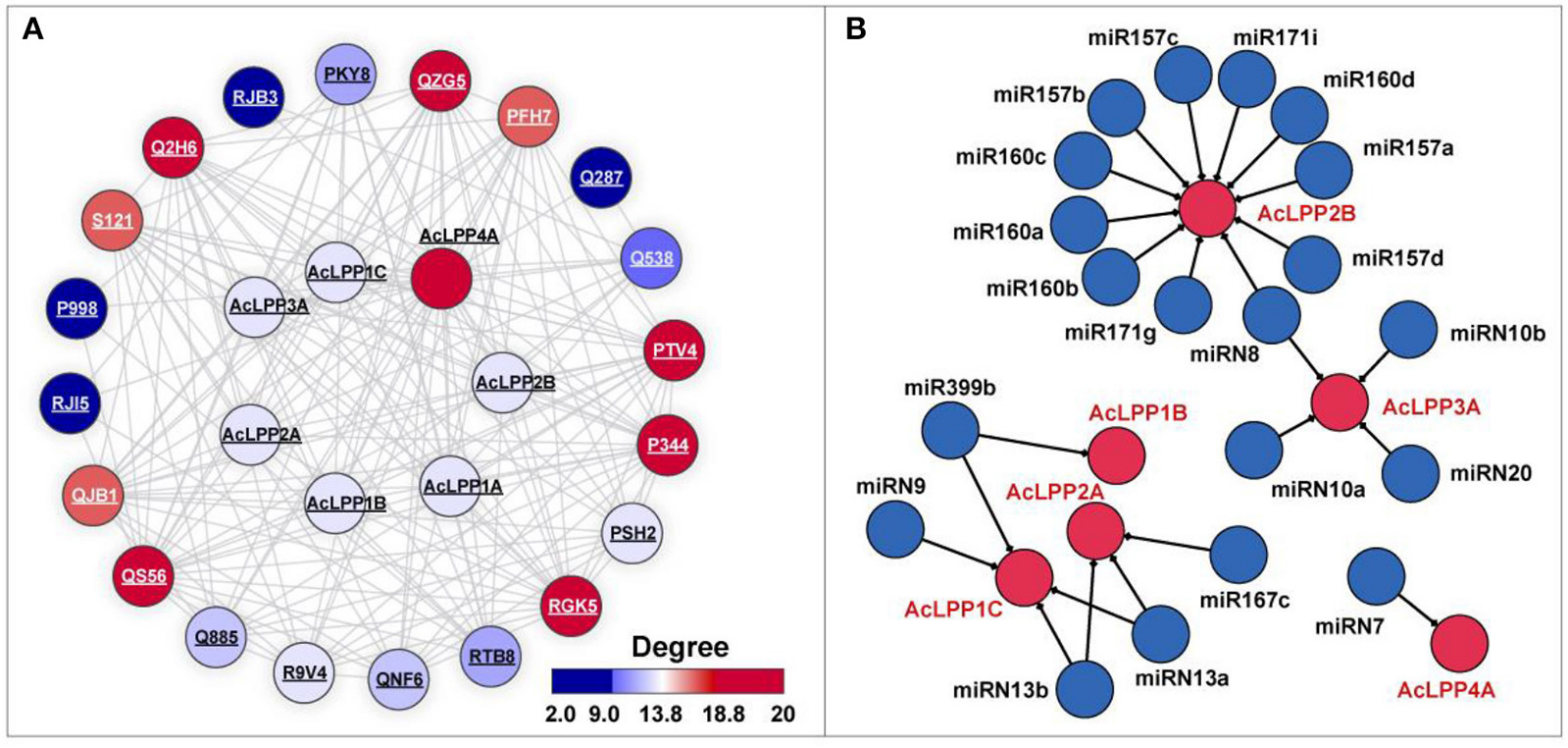
**(A)** Predicted protein-protein interaction networks of AcLPP proteins with other kiwifruit proteins using STRING tool. The protein node color represents the interaction degrees linked with each node. The two circles connected by the gray line represent the interaction between the proteins. **(B)** Schematic representation of the regulatory network relationships between the putative miRNAs and their targeted AcLPP genes. The miRNAs that regulate AcLPP gene are shown in blue circles. The miRNAs and AcLPP linked by the black lines indicate a putative regulatory relationship.

To reveal the comprehensive functions of AcLPPs, the PPI network of the AcLPP gene family was constructed. The result show that (Figure 3A) the AcLPP gene family mainly interacts with phospholipase and phospholipase hydrolase (Q885, R9V4, Q2H6, P344, PSH2, PTV4, QNF6, QZG5, RGK5) and glycerol-3-phosphate dehydrogenase (PKY8, P998, RJI5, RTB8). It was illustrated in the network (Figure 3A), the AcLPP4 protein had the most degrees, indicating that AcLPP4A might play a more important function. In addition, the proteins that AcLPP members contained were mainly related to phospholipids, which was also consistent with the function of cell component BP, which also confirms that AcLPP might interact with other proteins to participate in phospholipid-related biological processes.

In order to understand how AcLPP genes were regulated by miRNAs, putative miRNA-targeted *AcLPP*s were predicted revealing the regulating relationship (Figure 3B). The result showed that 6 AcLPP genes were predicted to be targeted by miRNA. AcLPP4A and AcLPP1B were targeted by one miRNA, AcLPP2B, AcLPP2A, AcLPP1C, and AcLPP3A were targeted by several miRNAs, of which AcLPP2B was targeted by 11 miRNAs. These results suggested that the AcLPP family was involved in complex regulatory networks of miRNA and played an important role in regulating the stress response of plants.

### Expression profile of AcLPP in different tissues, developmental stages, and postharvest storage

**Figure 4:**
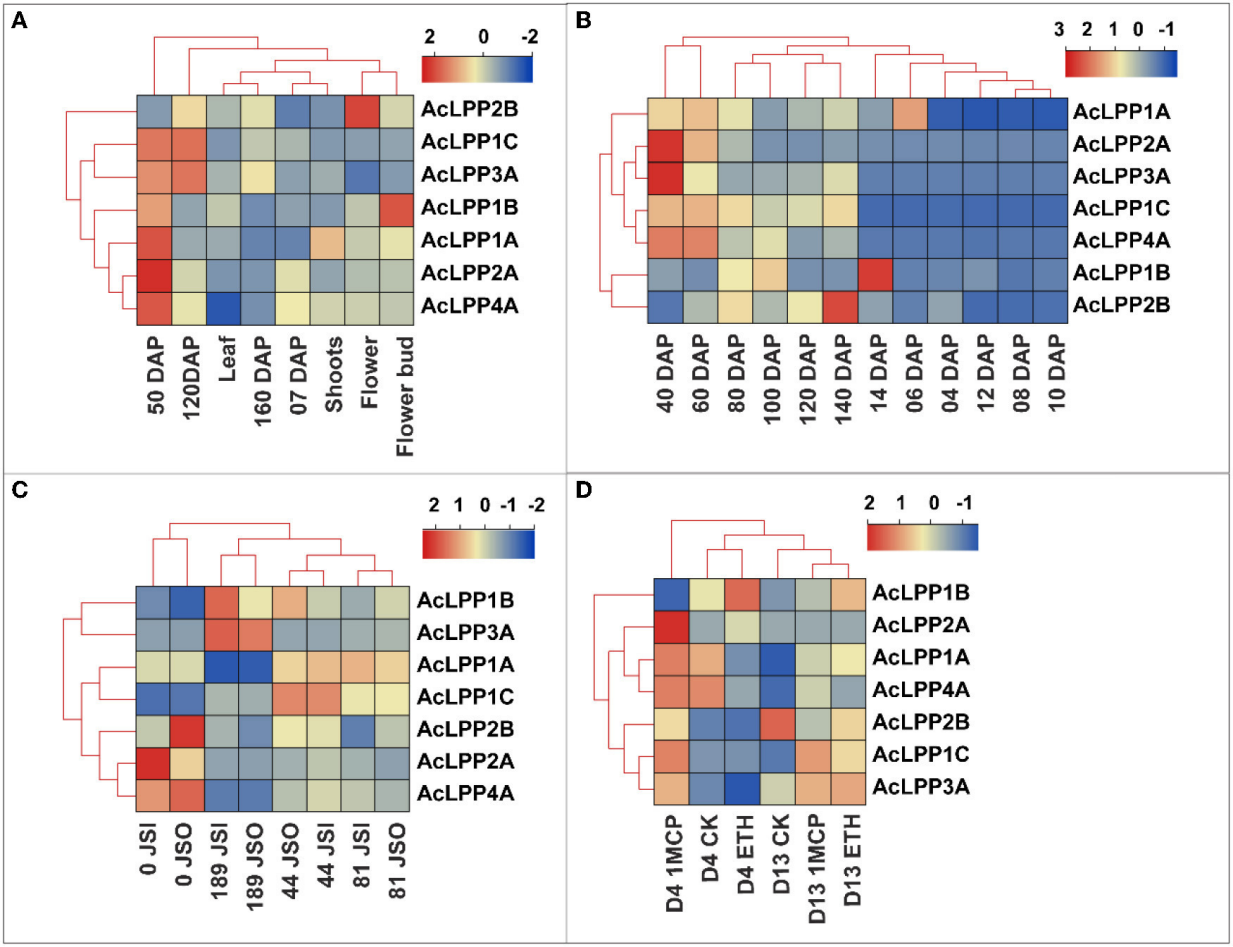
Expression profiles of AcLPP genes in different tissues, developmental stages and postharvest storage. **(A)** Expression of kiwifruit in stem, leaf and fruit tissues (DAP, days after pollination; DAH, days after harvest). **(B)** Expression of kiwifruit 40–140 days after pollination and 4–12 days of storage. **(C)** Expression of kiwifruit in mesocarp (JSO) and endocarp (JSI) after pollination. **(D)** Expression of kiwifruit under ethylene and 1-MCP Treatment. Different numbers represent days.

To understand the tissue-specific expression profile of AcLPP genes, FPKM (fragments per million mapped readings per thousand base transcripts) was used to evaluate their expression levels in different organs and stages of development. The RNA-seq data (PRJNA564374) of kiwifruit were extracted from the public database for analysis (Salazar et al., 2021). As shown in Figure 4A, *AcLPP2B* and *AcLPP1B* were highly expressed in flowers and flower buds, suggesting that *AcLPP2B* and *AcLPP1B* might coordinate the expression of target genes involved in flower development. Except for *AcLPP2B*, family members had relatively high expression in young fruits at 50 days after anthesis, while *AcLPP3A* and *AcLPP1C* were highly expressed in fruits at 120 days after anthesis. This indicated that family members might be involved in the process of fruit development. On the contrary, the expression level of family members in leaves and stems was relatively low.

The RNA-seq data (CRA003106) of kiwifruit during growth and development and postharvest storage showed that (Figure 4B) the *AcLPP2A* and *AcLPP3A* were highly expressed in young fruits at 40 days after anthesis, and *AcLPP2B* was highly expressed in young fruits at 140 days after anthesis. In addition, almost all members of the family were expressed in varying degrees during the growth and development of kiwifruit. During postharvest storage, *AcLPP1B* and *AcLPP1A* were highly expressed at 14 and 6 days after postharvest storage, indicating that these family members might be involved in some post-ripening stages of fruit softening.

Transcriptome data from pericarp and pulp (PRJNA625794) show that (Figure 4C): the expression of *AcLPP4A* and *AcLPP2A* were higher in the pulp of young fruit on the day 0 after anthesis, the expression of *AcLPP3A* and *AcLPP1B* were higher in the pulp 189 days after anthesis, and the expression of *AcLPP2B* and *AcLPP4A* were higher in the pericarp of the young fruit on day 0 after anthesis. In different stages of fruit development, the expression of AcLPP family members were different in pericarp and pulp, indicating that family members played different roles in different stages of fruit development.

Transcriptional data (PRJNA638129) of ethylene (ETH) and 1-methylcyclopropene (1-MCP) treatment of kiwifruit during storage are shown in Figure 4D: compared with the 4-day control, the family members (except *AcLPP1B*) had different degrees of high expression in 4d 1-MCP, and the expression level of 4d ETH treatment was lower than that of the control as a whole. Compared with the control, the expression of most members of the AcLPP family increased in ETH and 1-MCP on the 13d. This indicated that members of the AcLPP family were involved in the hormone response of ETH and 1-MCP, which might affect the softening process of kiwifruit.

### Expression pattern of AcLPP in hormone treatment and abiotic stress

**Figure 5:**
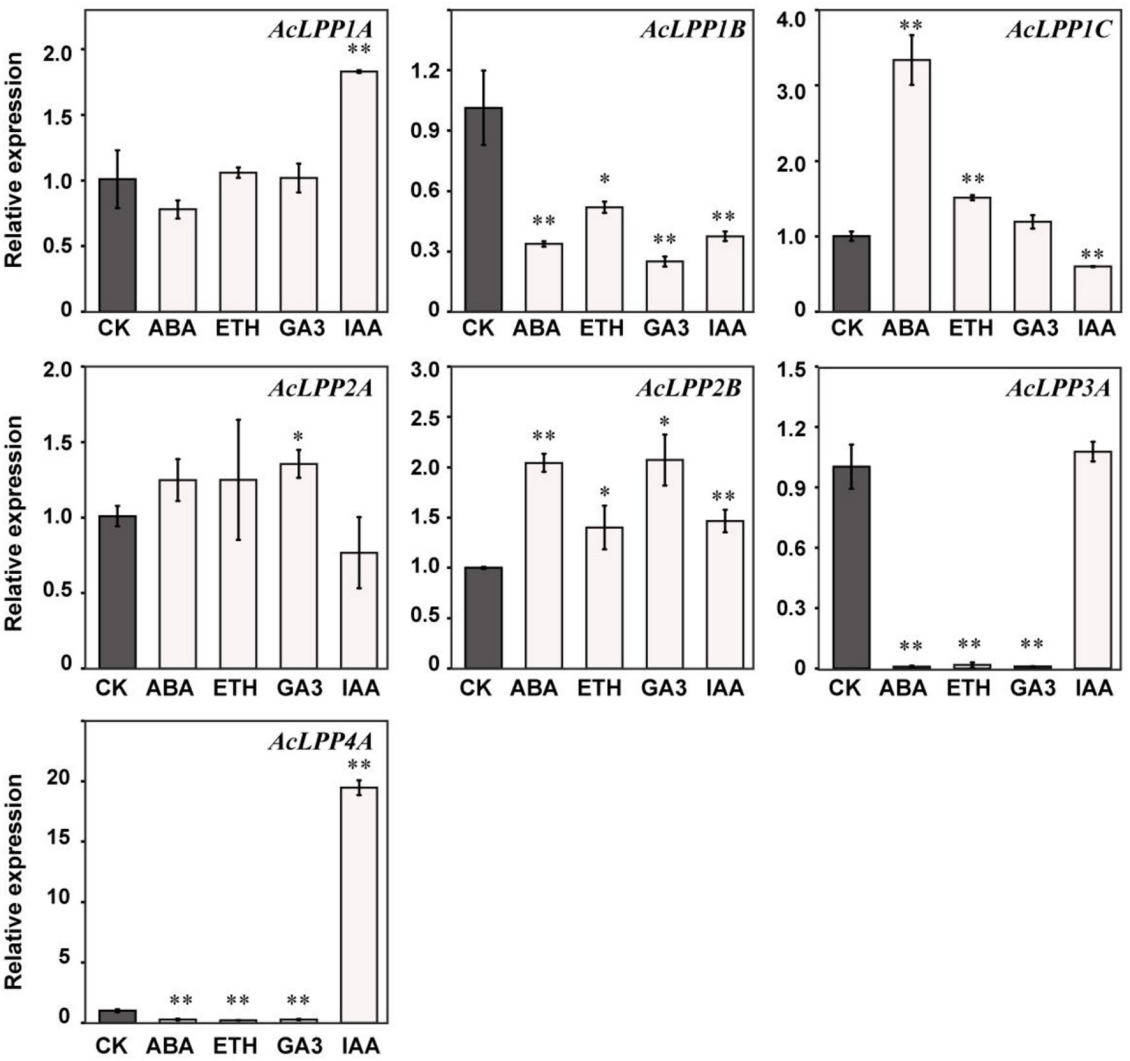
Expression analysis of 7 AcLPP genes in response to hormone treatments in parts of seedlings. Black columns stand for the expression levels of the plant leaf collected from kiwifruit seedlings, respectively. The X-axis represents various hormone treatments. CK, control sample; Eth, ethephon; GA_3_, gibberellin; IAA, indole acetic acids; ABA, abscisic acid. The expression data of control sample were normalized to 1. Error bars show the standard error between three replicates performed. Significance difference between treatment and the control were analyzed by *t*-test. *α = 0.05, **α = 0.01.

The regulatory role of plant hormones in all aspects of plant growth and development has been widely studied. To explore the relationship between AcLPP and hormone in kiwifruit, ethephon (ETH), gibberellin (GA_3_), auxin (IAA), and abscisic acid (ABA) were selected to treat kiwifruit seedlings, and the response expression of each of the *AcLPP* genes was observed. The result showed in Figure 5: under the treatment of abscisic acid (ABA), *AcLPP1*C and *AcLPP2B* were significantly up-regulated, they were up-regulated 3.3- and 2-folds, respectively. *AcLPP2A* performance was up-regulated but not significantly, and other members were down-regulated. Under ETH treatment, *AcLPP1C* and *AcLPP2B* were significantly up-regulated, *AcLPP1C* was upregulated 1.5-folds. while *AcLPP1B, AcLPP3A*, and *AcLPP4A* were significantly down-regulated. Under the treatment of gibberellin (GA_3_), *AcLPP2A* and *AcLPP2B* were significantly up-regulated, while *AcLPP1B, AcLPP3A*, and *AcLPP4A* were significantly down-regulated. Under the treatment of IAA. *AcLPP1A, AcLPP2B*, and *AcLPP4A* were significantly up-regulated, *AcLPP4A* was up-regulated by 20 times, while *AcLPP1B* and *AcLPP1C* were significantly down-regulated. There were significant differences in the expression levels of 7 *AcLPP* genes under different hormone treatments, which indicated that *AcLPP* genes responded to hormone regulation during plant development. It was also suggested that members of the *AcLPP* gene family in kiwifruit might be regulated by multiple hormone signals. Therefore, it was reasonable to believe that some *AcLPP* genes might be involved in the interaction between different plant hormones, which was a good reference for us to study the field of plant hormone interaction in the future.

**Figure 6:**
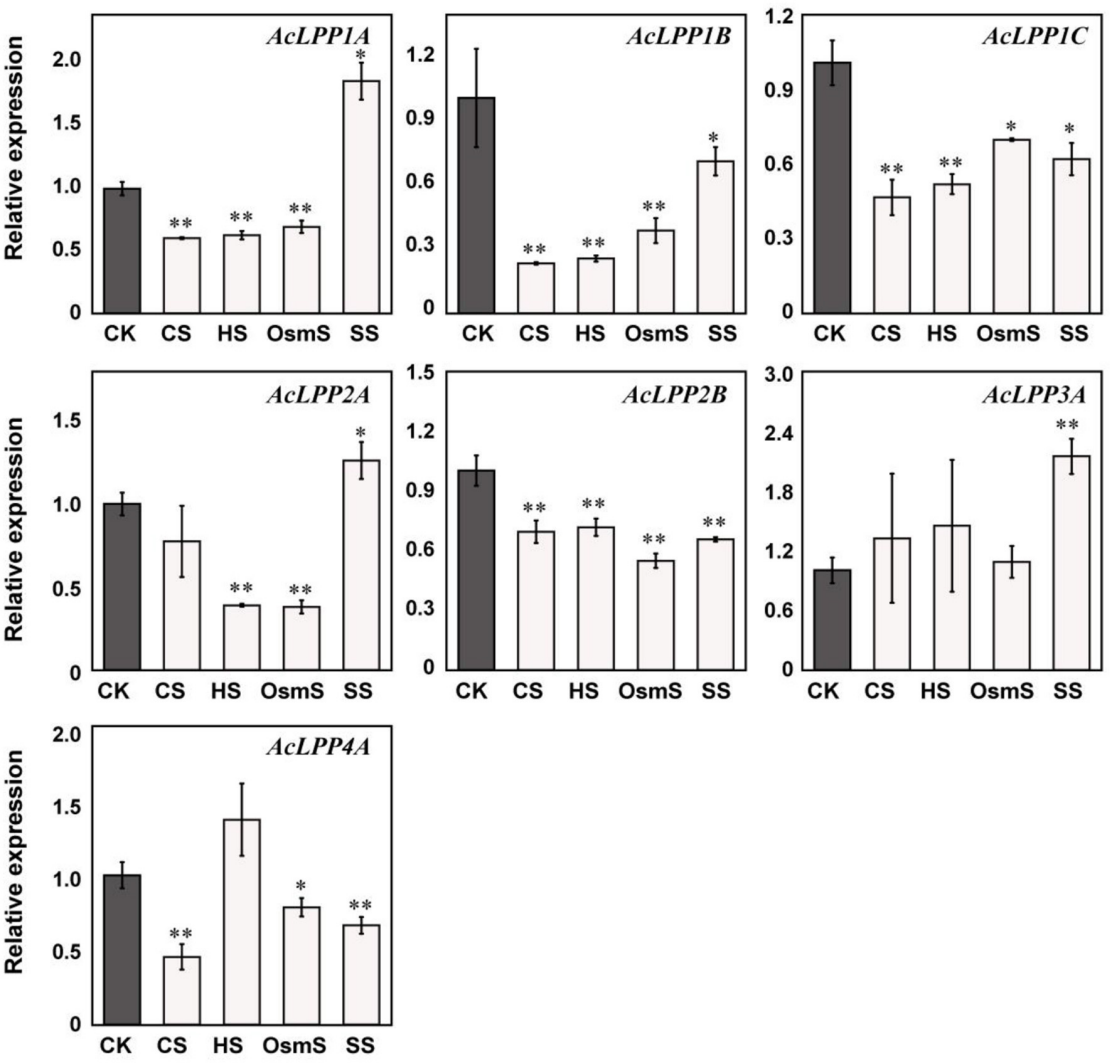
Expression analysis of 7 AcLPP genes in response to abiotic treatments. The X-axis represents different abiotic stresses. CK, control sample; SS, salt stress; CS, cold stress; HS, heat stress; OsmS, osmotic stress. The expression data of control sample were normalized to 1. Error bars show the standard error between three replicates performed. Significance difference between treatment and the control were analyzed by *t*-test. *α = 0.05, **α = 0.01.

In order to explore the involvement of *AcLPP* genes in plant defense under abiotic stress, the gene expression of family members under salt stress, osmotic stress, cold stress, and heat stress were analyzed. The result showed that (Figure 6): Under cold stress (CS), family members except AcLPP2A and AcLPP3A were significantly down-regulated; under heat stress (HS), members except *AcLPP3A* and *AcLPP4A* were also significantly down-regulated, *AcLPP3A* and *AcLPP4A* were up-regulated 1.45- and 1.37-folds, and under osmotic stress (OsmS), family members were down-regulated, of which 6 members were significantly down-regulated. Under salt stress (SS), *AcLPP1A, AcLPP2A*, and *AcLPP3A* were significantly up-regulated, while other members were significantly down-regulated. Members of the *AcLPP* gene family showed sensitivity response to all stress conditions, suggesting that these genes were involved in regulating stress response.

## Discussion

So far, LPP family genes have been reported in different plant species, such as *Arabidopsis* (Paradis et al., 2011), barley (Barrero et al., 2009), cowpea (Franca et al., 2008), tobacco (Pleskot et al., 2012), and *B. rapa* (Su et al., 2021). However, less research has been carried out to comprehensively identify and characterize the LPP genes in kiwifruit, which is an important economic fruit. In this study, 7 AcLPP genes were identified in the kiwifruit genome. According to the similarity of protein sequences and their phylogenetic relationship,7 AcLPP were divided into 4 subgroups (Figure 2B). Gene structure and motif studies indicated relative conservation among members of the same subfamily (Figure 1A and Supplementary Table 2). Typical features of the LPP structure include: transmembrane domain, conserved PAP2 domain, and functional domains associated with catalytic activity, Transmembrane domains are typical of membrane proteins and play an important role in transducing transmembrane signals. Several transmembrane domains were also found in the kiwifruit LPP genes structure, this is consistent with what has been reported for *Arabidopsis* (Paradis et al., 2011) and tobacco (Pleskot et al., 2012). However, the domains of different AcLPP members vary, implying possible differences in their functions. We also found similar sequences within the same subfamily of the LPP genes family in the evolutionary tree analysis (Supplementary Table 6), which indicates that similar evolutionary events have taken place among the 5 species.

Almost all AcLPP were located on the plasma membrane, except that AcLPP2A and AcLPP4A were specifically located in the endoplasmic reticulum and cytoskeleton. In *Arabidopsis thaliana*, AtLPP2 was localized to the cell membrane to function (Katagiri et al., 2005). In rapeseed, 10 BnLPP proteins were localized in the plasma membrane (Su et al., 2021). These results were consistent with previous studies that all LPP, as liposoluble proteins, are mainly localized on the plasma membrane (Kok et al., 2012).

The analysis of *cis*-acting elements analysis showed that *AcLPP* genes may respond to different stress and hormone signals (Figure 1B). We also found many *cis*-elements that were commonly associated with defense and stress responsiveness, phytohormone response, and light response. The *AcLPP* genes contain methyl jasmonate response (MeJA) *cis*-elements (TGACG-motif and CGTCA motif), auxin responsive element (TGA-box), abscisic acid responsive element (ABRE), and light responsive element (box-4, G-box, MBS and GT1 motif). Related studies show that light signaling can be modulated by ethylene and growth hormone in tomato fruit metabolism (Cruz et al., 2018). Research shows that TGACG-motif, TGA-element, ABRE, G-box, MBS and GT1-motif have regulatory effects under salt stress (Yamniuk and Vogel, 2004). Studies have shown that these cis-acting elements are able to respond to changes in external stimuli and rapidly regulate gene expression (Zhou et al., 2021). These findings provide a basis for our in-depth understanding of the molecular mechanisms underlying the function of the *AcLPP* genes. Therefore, the expression patterns of *AcLPP* genes under ETH, GA_3_, IAA, and ABA treatments were studied. Among the four plant hormones studied, IAA is involved in almost all aspects of plant growth and development (Woodward and Bonnie, 2005). IAA and GA_3_ play a unique role in cell division and expansion during fruit setting and in the later stage. They regulate pollination and fertilization together through crosstalk (Li et al., 2016). Ethylene and abscisic acid play an important role in plant senescence and biological stress (Fujita et al., 2006). The results showed that the expression levels of most *AcLPP* genes varied greatly under different hormone treatments, mainly down-regulated and few up-regulated genes, among which *AcLPP1B* was significantly down-regulated under different hormone treatments. This indicates that the *AcLPP* gene responded to hormonal and abiotic stress processes. Under abiotic salt stress, osmotic stress, cold stress, and heat stress, many *AcLPP* genes could be induced by more than one stress, which also confirmed that kiwifruit *AcLPP* family members were widely involved in the process of adaptation to the environment.

Gene replication events are an important source of the expansion of plant gene families (Blanc and Wolfe, 2004). In this study, 5 pairs of fragment duplications and 1 pair of tandem duplications (AcLPP1B&AcLPP2A) were found in the members of the kiwifruit LPP family. Studies show that segmental, tandem, and whole-genome duplications (WGD) caused the expansion of gene families primarily by factors (Freeling, 2009). The results indicated that gene duplication was the main driving force for the expansion of the kiwifruit LPP family. In addition, major drivers of the evolution and expansion of gene families such as GmARF, GmHD-Zip, and GmMYB were segmental duplications (Du et al., 2012; Chen et al., 2014; Le et al., 2016). Our results demonstrated that all duplicated gene pairs possess Ka/Ks < 1. such as, the Ka/Ks < 1 had been reported SWEET genes family (Patil et al., 2015), GRAS genes family and Aux/IAA (Singh and Jain, 2015). These results indicate that the *AcLPP* gene family had evolved primarily through positive selection and the conserved AcLPP proteins evolve more rapidly at the protein level. To have a deeper understanding of the evolutionary relationship of LPP genes, comparative genomics was used to analyze the evolutionary events of LPP genes (Figure 4). The results revealed that the LPP genes of kiwifruit were more related to the monocotyledon *Arabidopsis* and *B. rape*. The collinearity relationship with the model plant tomato was not found in the collinearity module. It could be inferred that the macaque picked *AcLPP* gene and *Arabidopsis* and *B. rape* might originate from the same ancestor, and differentiation occurs during the process, and the function was conservative and important. It has been shown that genes containing fewer introns are transcribed more rapidly (Jeffares et al., 2008). Therefore, genes containing fewer introns in the *AcLPP* gene might respond rapidly transcriptionally to stress and abiotic stress. Many studies have shown that during evolution, introns have been inserted and retained in plant genomes (Rogozin et al., 2003). Therefore, we speculate that the increase or loss of subfamily specificity of introns in the LPP coding region might explain the closely related functional differences between *AcLPP*. In addition, in the conservative motif analysis, the structural diversity of *AcLPP* was strongly supported by the functional diversity of AcLPP.

MiRNA is a class of non-coding single-stranded RNA molecules with a length of approximately 22 nucleotides encoded by endogenous genes. They were involved in post-transcriptional gene expression regulation in animals and plants (Wang et al., 2018). Presumably miRNAs that regulate AcLPP genes in kiwifruit, 6 AcLPP genes were regulated by multiple miRNAs. In addition, the AcLPP protein interaction network further revealed that AcLPP mainly interacts with phospholipid-related proteins to function. The regulation between miRNA-miRNA and the integration of the protein interaction network would help to better understand the function of kiwi AcLPP gene and pave the way for future research.

## Conclusion

The present study is a comprehensive and systematic report for the characterization of AcLPP family genes. A total of 7 *AcLPP* were identified, the phylogeny, chromosome distribution, gene structure, conserved motif, *cis*-element, and expression analysis were helpful to study the function of the LPP gene family. The expression profiles of *AcLPP* in different tissues, developmental stages and postharvest storage revealed that some members of the LPP gene family were involved in some stages of growth, development, and post-ripening of kiwifruit, and there were significant differences in the expression levels of 7 *AcLPP* genes under four different hormone treatments, which indicated that the *AcLPP* gene responded to the hormonal regulation of plant development. Family members showed sensitive responses to four kinds of abiotic stress conditions, suggesting that these genes were involved in regulating stress response. This preliminary analysis found that *AcLPP* genes may be involved in plant hormone signal interaction and stress response. In a word, these results can and should provide a basis and reference for studying agricultural characters and improving the stress resistance of kiwifruit.

## Data availability statement

The datasets presented in this study can be found in online repositories. The names of the repository/repositories and accession number(s) can be found in the article/Supplementary material.

## Author contributions

XR and YD designed the experiments. YY, LC, GS, and FL carried out the experiments and performed the data processing. GC, QZ, and RL contributed to analysis and interpretation of data. LX, YY, and YD wrote and revised the article. XR directed the study. All authors contributed to the article and approved the submitted version.

## Conflict of interest

The authors declare that the research was conducted in the absence of any commercial or financial relationships that could be construed as a potential conflict of interest.

## Publisher's note

All claims expressed in this article are solely those of the authors and do not necessarily represent those of their affiliated organizations, or those of the publisher, the editors and the reviewers. Any product that may be evaluated in this article, or claim that may be made by its manufacturer, is not guaranteed or endorsed by the publisher.
