# Supplementary material for: Identification and expression analysis of the lipid phosphate phosphatases gene family reveal their involvement in abiotic stress response in kiwifruit
Source: Front Plant Sci. 2022 Aug 24;13:942937. doi: 10.3389/fpls.2022.942937 (PMC9449726; doi:10.3389/fpls.2022.942937)
Supplement: Supplementary file 6 [file Table_6.docx]

**Table S6. The protein sequences of LPP gene family in A. thaliana, B. oleracea, B. rapa ,B. napus and kiwifruit**

>AtLPP4

MAKIMLGSHSVKSHGWKVAREHLCDWLILVVLGLIDIVLNVIEPFHRYIGPDMLTDLTFP

FYEDTIPMWAVPIICILVPICIFIVYYYYRRDVYDLHHAILGIGFSCLVTGVTTDSIKDA

VGRPRPNFFYRCFPNGKPKFHPDTKDVVCHGVKKIIKEGYKSFPSGHTSWSFAGLTFLAW

YLSGKIKVFDRRGHVAKLCLVFLPILISILIGISRVDDYWHHWTDVFAGAIIGIFVASFS

YLHFFPYPYDENGWAPHAYFRMLAERSTGRATTMTRTGSRGMLGNDVEPGNSASSPHDRH

RESTDSDF

>AtLPP3

MARFSFPCFPNFGGFNQAVTNRGPEISETADNWVSPSDIPLIEPNSKEHRMREAQLGGHT

LRSHGMTVARTHMHDWIILVLLVILECVLLIIHPFYRFVGKDMMTDLSYPLKSNTVPIWS

VPVYAMLLPLVIFIFIYFRRRDVYDLHHAVLGLLYSVLVTAVLTDAIKNAVGRPRPDFFW

RCFPDGKALYDSLGDVICHGDKSVIREGHKSFPSGHTSWSFSGLGFLSLYLSGKIQAFDG

KGHVAKLCIVILPLLFAALVGISRVDDYWHHWQDVFAGGLLGLAISTICYLQFFPPPYHT

EGWGPYAYFQVLEAARVQGAANGAVQQPPPQVNNGEEEDGGFMGLHLVDNPTMRREEDVE

TGRG

>AtLPP2

MPEIHLGAHTIRSHGVTVARFHMHDWLILLLLIVIEIVLNVIEPFHRFVGEDMLTDLRYP

LQDNTIPFWAVPLIAVVLPFAVICVYYFIRNDVYDLHHAILGLLFSVLITGVITDAIKDA

VGRPRPDFFWRCFPDGIGIFHNVTKNVLCTGAKDVVKEGHKSFPSGHTSWSFAGLGFLSL

YLSGKIRVFDQRGHVAKLCIVILPLLVAALVGVSRVDDYWHHWQDVFGGAIIGLTVATFC

YLQFFPPPYDPDGWGPHAYFQMLADSRNDVQDSAGMNHLSVRQTELESVR

>AtLPP1

MTIGSFFSSLLFWRNSQDQEAQRGRMQEIDLSVHTIKSHGGRVASKHKHDWIILVILIAI

EIGLNLISPFYRYVGKDMMTDLKYPFKDNTVPIWSVPVYAVLLPIIVFVCFYLKRTCVYD

LHHSILGLLFAVLITGVITDSIKVATGRPRPNFYWRCFPDGKELYDALGGVVCHGKAAEV

KEGHKSFPSGHTSWSFAGLTFLSLYLSGKIKAFNNEGHVAKLCLVIFPLLAACLVGISRV

DDYWHHWQDVFAGALIGTLVAAFCYRQFYPNPYHEEGWGPYAYFKAAQERGVPVTSSQNG

DALRAMSLQMDSTSLENMESGTSTAPR

>AcLPP1A

MAWRNMLSFCSFPNFWRNFQGRVSEVELGAHTVKSHGTTVARNHMHDWLILLLLGVIEII

LYIIHPFYRYVGEHMMTDLKYPLKDNTVPVWAVPMYAVLLPIVIFVLFYFRRRDVYDLHH

SILGLLFAVLITGVLTDAIKNAVGRPRPDFFWRCFPDGIDNYDKWGNVICHGKDSVIKEG

HKSFPSGHASWSFAGLGFLSLYLSGKIKAFDRRGHVAKLCIVLLPLLVASLVAISRVDDY

WHHWQDVFAGGLLGLVVSTFCYLQFFPPPYQNEGWGPYAYFRALEESHANSHLGHPVGES

NVQFTEAQVVNQQTDRDNHGLISGAVRNSSSMDDLESGRR

>AcLPP2A

MPDIQIAHTIRSHGAQVVRAHMHDWLILLLLVVVEVILNLIEPFHRFVGEEMLTDLKYPM

KGNTVPFWAVPVIAILVPLVVILVYYFIRWDVYDFHQAILGLLFSVLITGVITDAIKDAV

GRPRPDFFWRCFPNGKGVFNNVTTNVVCTGKKSDIMEGHKSFPSGHTSWSFAGLGFLSWY

LSGKIRAFDRRGHVSKLCIILLPLLVAALVGVSRVDDYWHHWQDVFAGGFLGLTVASFCY

LQFFPPPYDIDGWAPHSYFQMLAESQHGNQTLSSRINHLVTPPTERENVYIQSHQGMAMS

DINTWDTSPILNEMERGQICRL

>AcLPP1B

MAWKNMICSCSFLNFRSIFQGGRMSVEELDAHTIRSHGATVARKHMHDWLILLLLVVIEI

ILNIIHPFYRFVGEHMMTDLKYPMKDNTVPVWAVPMYAVLLPVVVFLFFYFRRRCVYDLH

HAILGLLFAVLITGVLTDSIKNAVGRPRPDFFWRCFPDGVDSYDKWGNVVCHGEDSVIKE

GHKSFPSGHTSWSFAGLGFLSLYLSGKIKAFDRKGHVAKLCIVFLPLLVASLVGISRVDD

YWHHWQDVFAGGLLGLFVATFCYLQFFPPPYHTDGWGPYEYFSVQEQSRLNPHLSHPANG

SNAQAQEAQALNQETGRHTSSMDDIESGRK

>AcLPP4A

LAKMAVTQYGSYTIKSHGGKLARTHMHDWLILLVLALIIGALNYIEPFHRFVGKEMMTDL

KFPFKPDTIPFWTVRVYVGLFPCVVFLVYYISRRDIYDLHHAVLGLLLSVLITGVITDSI

KDAVGRPRPNFYWRCFVDGVSVFDPSTMDVMCTNKNLKVTKEGYKSFPSGHTSWSFAGLG

FLSLYLSGKIRAFNRNGHISKLCFVILPYLLAALVGVSCVDDYWHHWTDVFTGAVIGTVV

SALCYLHFFPSPHCTNSWAPYAYFRILAESGVQLSPAEMEAPYARHGYDEETGESSSLSA

GQDLEELEAGKKH

>AcLPP3A

MMEVQPETHTLRSHGVTVARTHVHDWLISMLLVVIYVMLNLIHPFHRFVGKDMMDDLKYP

LKDNTVPSWAVPIHAVLLPMAIFLFFYFRRRDVYDLHHAILGLLFSVLVTAVITSALKDA

VGRPRPNFFWRCFPDGKDVYDQWGNVICHGKQSGVQEGYKSFPSGHTSWSFAGLGFLSLY

LSGKIKAFDRRGHVAKLCVVFLPLLVASLVAISRINDYWHHWEDVFAGGLLGLTVATFCY

LQFFPPPYHVEGWGPYAYFRVLEESRAATQPANAVNALNVRSTEARMESQQDERNINSFT

GISLAGDSSLTAEDLESGR

>AcLPP1C

MAWKNLICFRSFPNFRGIFQGRVSGEELDAHTIRSHGVTVVRKHKYDWLILLLLVVIVII

LNFINPFYRFVGEDMMTDLKYPMKDNTVPVWAVPMYAVLLPIVVFLAFYFRRRCVYDLHH

AILGLLFAVLITGVLTESIKNAVGRPRPDFFWRCFPDGIDNYDKWGNVVCHGEDSIIKEG

HKSFPSGHTSLSFTGLGFLSLYLSGKIKAFNRKGHVAKLCIVFLPLLVASLVGISRVDDY

WHHWQDVFAGGLLGFVVATFCYLQFFPPPYHTDGWGPYAYFRAQEESRLNPQLGQPANGP

NVQAQALNQETGGRYTSSMDDIESGRK

>AcLPP2B

MPEIQLGAHTLRSHGAKVARIHMHDWLILLLLVVIEVILNLIEPFHRFVGEEMMEDLKYP

LQGNTVPIWAVPILGILLPLAIILTYYIIRRDVYDFHHAMLGLLFSVLITGVITDAIKDA

VGRPRPDFFWRCFPDGKGVFDPVTRNVMCTGLKSVIKEGHKSFPSGHTSWSFAGLGFLSL

YLSGKIRAFDRGGHVAKLCIIVLPLLVASLVGVSRVDDYWHHWQDVFAGGLLGLTVASFC

YLQFFPPPYDVDGWGPHAYFEMLADSRNGTQSSANSTSCLIVCQTELDNVYAQSQHGMSS

SEVRTRDTSPILDEMESGRRH

>BoLPP1

MVLGSLFAPLKFWGNTQEQEPQRGRMQEIDLGIHTIRSHGGGLASKHKHDWIILVILVAIEIGLQLISPFYRYVGKDMMTDLKYPFNDNTIPVWSVPIYAVLLPIIIFVCFYVKRTCVYDLHHSILGLLFTVLITGIITDSIKLATGRPRPNFYWRCFPDGKELYDALGGVVCHGEPGEVKEGHKSFPSGHTSWSFAGLGFLSLYLSGKIKAFNREGHVAKLCIVFAPLLAACLVGISRVDDYWHHWQDVFAGGLIGLFVAAFCYRQFYPNPYHEEGWGPYAYFIAALERGSQQNGDALRTMSLQVEPTSLENMESGTSTAPR

>BoLPP2

MGKITLGSHTVKSHGWKVAREHLIDWIILVLLGIVIIVLNIMEPFHRYIGPDMLTDLTFPLYQDTIPIWAVPIICILVPICIFTVYYYFGRDVYDLHHAILGIGFSCLVTGVTTDSIKNAVGRPRPNFFHRCFPDGKAKFDSVTKDVVCHGVKKIIKEGYKSFPSGHTSWSFAGLTFLAWYLSGKIKVFDRRGHVAKLCLVFLPILVAVLIGITRVDDYWHHWTDVFAGAIIGIFFSSLSYLHFFPYPYDDNGWAPHAYFRMLAESSRRDTTMTRSGSREMLENDVELGSSSMPHHRNRGSTDSE

>BoLPP3

MQEIDLGIHTIRSHGGSVASKHKHDWIILVILVAIEIGLQLISPFYRYVGRDMMTDLKYPFNDNTVPVWSVPIYAVLLPIIIFVCFYMKRTCVYDLHHSILGLLFTVLITGIITDSIKLATGRPRPNFYWRCFPDGKEVYNALGGVICHGKPGEVKEGHKSFPSGHTSWSFAGLGYLSLYLSGKVKAFNREGHVAKLCIVFAPLLAACLVGISRVDDYWHHWQDVFAGALIGLFVAAFCYRQFYPNPYHEEGWGPYAYFRAVQERGQAQNGDALRTMSLQVEPTSLENMESGTSSVRR

>BoLPP4

MPEIQLGAHTIRSHGVAVARFHMHDWLILVLLIVIEVVLNVIEPFHRFVGEDMLTDLRYPLQDNTVPFWAVPLIAVVLPFAVISVYYFIRRDVYDLHHAILGLLFSVLITGVITDAIKDAVGRPRPDFFWRCFPDGRGRGHVAKLCIVFLPLLVAALVGVSRVDDYWHHWQDVFGGAIIGLTVASFCYLQFFPPPYDPDGWGPHAYFQMLADSRNVVQNSAGMNNLSVRQAELENVYVDQQGTSMEISRSNTRDTTRTLESC

>BraLPP1

MMREAHQSTHTVRSHGMTLARTHMHDWIILVLLVILECILLIIHPFYRFVGKDMMTDLSYPLKSNTVPIWSVPVYAMLLPLAIFIFIYFRRRDVYDLHHAVLGLLYSVLVTAVLTDSIKNAVGRPRPDFFWRCFPDGKAVYDALGDVICHGDKSVIREGHKSFPSGHTSWSFAGLGFLSLYLSGKIRAFDGKGHVAKLCIVILPLLVAALVGISRVDDYWHHWQDVFAGGLLGIVVSTFCYLQFFPPPYRTEAWGPYAYFLVLEAARAQAQAAENEMAQRPPQGDNGEEEDGGFMGLHLVDNPSMRREEADVEAGRVPSRG

>BraLPP2

MGKITLGSHTVKSHGWKVAREHLIDWIILVLLGIVIIVLNIMEPFHRYIGPDMLTDLTFPLYQDTIPIWAVPIICILVPICIFTVYYYFGRDVYDLHHAILGIGFSCLVTGVTTDSIKNAVGRPRPNFFHRCFPDGKAKFDSVTKDVVCHGLKKIIKEGYKSFPSGHTSWSFAGLTFLAWYLSGKIKVFDRRGHVAKLCLVFLPILVAVLIGITRVDDYWHHWTDVFAGAIIGIFFASLSYLHFFPYPYDDNGWAPHAYFRMLAESSRRDTTMTRSSSREMLENDVELGSTSMPHHRNRGSTDSE

>BraLPP3

MGIVIVAVTNRGPEVSGTADNWVSPSDIPLIEPYSKEHKMREAQLGAHTVRSHGMTLARTHMHDWIILVLLVILECILLIIHPFYRFVSKDMMTDLSFPLKSNTVPIWTVPIYAMLLPLVIFIAIYFRRRDVYDLHHAVLGLLYSVLVTAVLTDSIKNAVGRPRPDFFWRCFPDGKAVYDSLGDVICHGDKSVIREGHKSFPSGHTSWSFAGLGFLSLYLSGKIQAFDGKGHVAKLCIVILPLLVAALVGISRVDDYWHHWQDVFAGGLLGLVVSTFCYLQFFPPPYHTEAWGPYAYFQVLEAARAQAQAAENGVAQGGNGEEEDGGFMGLHLVDNPSMRREDEDVEAGRVPRG

>BraLPP4

MYFCKLRKDSDESYRQNWLDVKQSVVVEQEPQRGRMQEIDLAIHTIRSHGAGVASKHKHDWIILVILVAIEIGLQLISPFYRYVGRDMMTDLKYPFNDNTVPVWSVPIYAVLLPIIVFVCFYMKRTCVYDLHHSILGLLFTVLITGIITDSIKLATGRPRPNFYWRCFPDGKEVYNALGGVICHGKPGEVKEGHKSFPSGHTSWSFAGLGYLSLYLSGKVKAFNREGHVAKLCLVFAPLLAACLVGISRVDDYWHHWQDVFAGALIGLFVAAFCYRQFYPNPYHEEGWGPYAYFRAAQERGQAQNGDVLRTMSLQVEPTSLENMESGTSSVRR

>BraLPP5

MPEIQLGAHTIRSHGVTVARFHMHDWLILLLLIVIEVVLNVIEPFHRFVGEDMLTDLRYPLQDNTVPFWAVPLIAVVLPFVVISVYYFIRRDVYDLHHAILGLLFSVLITGVITDAIKDAVGRPRPDFFWRCFPDGRGFFHNVTRDVLCTGDKNVVKEGHKSFPSGHTSWSFAGLGFLALYLSGKIRVFDQRGHVAKLCIVFLPLLVAALVGVSRVDDYWHHWQDVFGGAIIGLTVASFCYLQFFPPPYDPDGWGPHAYFQMLADSRNVVQNSAGMNNLSVRQAELENVYVHQQGTSMEISRSNTRGTPQEC

>BraLPP6

MLKILSPAPRLCCPGIAFSCLVTGVTTDSIKDAVDWPRPNFFYRCFPDGKPKFDPETRMLYHGVKKIIKEGYKSFPSGHTSWWAPHAYFRMLAEKSGLATTMTHGAVLE

>BnLPP1.1

MNCIPLLRSSKSETSNPINLQEQEPQRGRMQEIDLGIHTIRSHGVGVASKHKHDWIILVILVAIEIGLQLISPFYRYVGRDMMTDLKYPFNDNTVPVWSVPIYAVLLPIIVFVCFYMKRTCVYDLHHSILGLLFTVLITGIITDSIKLATGRPRPNFYWRCFPDGNEVYNALGGVICHGKPGEVKEGHKSFPSGHTSWSFAGLGYLSLYLSGKVKAFNREGHVAKLCLVFAPLLAACLVGISRVDDYWHHWQDVFAGALIGLFVAAFCYRQFYPNPYHEEGWGPYAYFRAAQERGQAQNGDVLRTMSLQVEPTSLENMESGTSSVRR

>BnLPP1.2

MVFGSFFSSLKFWGNSQEQEPQRGRMQEIDLGIHTIRSHGGSVASKHKHDWIILVILVAIEIGLQLISPFYRYVGRDMMTDLKYPFNDNTVPVWSVPIYAVLLPIIIFVWFYMKRTCVYDLHHSILGLLFTVLITGIITDSIKLATGRPRPNFYWRCFPDGKEVYNALGGVICHGKPGEVKEGHKSFPSGHTSWSFAGLGYLSLYLSGKVKAFNREGHVAKLCIVFAPLLAACLVGISRVDDYWHHWQDVFAGALIGLFVAAFCYRQFYPNPYHEEGWGPYAYFRAVQERGQAHNGDALRTMSLQVEPTSLENMESGTSSVRR

>BnLPP1.3

MVLGSLFAPLKFWGNTQEQEPQRGRMQEIDLGIHTIRSHGGGLASKHKHDWIILVILVAIEIGLQLISPFYRYVGKDMMTDLKYPFNDNTIPVWSVPIYAVLLPIIIFVCFYVKRTCVYDLHHSILGLLFTVLITGIITDSIKLATGRPRPNFYWRCFPDGKELFDALGGVVCHGEPGEVKEGHKSFPSGHTSWSFAGLGFLSLYLSGKLKAFNREGHVAKLCIVFAPLLAACLVGISRVDDYWHHWQDVFAGGLIGLFVAAFCYRQFYPNPYHEEGWGPYAYFIAAQERGQQQNGDALRTVSLENMESGTSTAPR

>BnLPP2.1

MPEIQLGAHTIRSHGVTVARFHMHDWLILVLLIVIEVVLNVIEPFHRFVGEDMLTDLRYPLQDNTVPFWAVPLIALVLPFAVISVYYFIRRDVYDLHHAILGLLFSVLITGVITDAIKDAVGRPRPDFFWRCFPDGRGFFHNVTRDVLCTGDKDVVKEGHKSFPSGHTSWSFAGLGFLALYLSGKIRVFDQRGHVAKLCIVFLPLLVAALVGVSRVDDYWHHWQDVFGGAIIGLTVASFCYLQFFPPPYDPDGWGPHAYFQMLADSRNVVQNSAGMNNLSVRQAELENVYVDQQGTSMEISRSNTRDTTRTLESC

>BnLPP2.2

MPEIQLGAHTIRSHGVTVARFHMHDWLILLLLIVIEVVLNVIEPFHRFVGEDMLTDLRYPLQDNTVPFWAVPLIAVVLPFVVISVYYFIRRDVYDLHHAILGLLFSVLITGVITDAIKDAVGRPRPDFFWRCFPDGRGFFHNVTRDVLCTGDKDVVKEGHKSFPSGHTSWSFAGLGFLALYLSGKIRVFDQRGHVAKLCIVFLPLLVAALVGVSRVDDYWHHWQDVFGGAIIGLTVASFCYLQFFPPPYDPDGWGPHAYFQMLADSRNVVQNSAGMNNLSVSQAELENVYVHQQGTSMEISRSNTRGTPQEC

>BnLPP3.1

MPFPRFLNFGGLFQAVTNREPEISVTADNWVSPSDIPLIEPFSKEHKMREAQLGAHTVRSHGMTLARTHMHDWIILVLLVVLECILLIIHPFYRFVGKDMMTDLSYPLKSNTVPIWSVPVYAMLLPLVIFISIYFRRRDVYDLHHAVLGLLYSVLVTAVLTDSIKNAVGRPRPDFFWRCFPDGKAVYDTLGDVICHGDKSVIREGHKSFPSGHTSWSFAGLGFLSLYLSGKIQAFDGKGHVAKLCIVILPLLVAALVGISRVDDYWHHWQDVFAGGLLGLVVSTFCYLQFFPPPYHTEAWGPYAYFQVLEAARAQAQAAENGVAQGGNGEEEDGGFMGLHLVDNPSMRREEEDVEAGRVPRG

>BnLPP3.2

MPFPRFLNFGGIFQAVTNRGPEVSGTADNWVSPSDIPLIEPYSKEHKMREAQLGAHTVRSHGMTLARTHMHDWIILVLLVVLECILLIIHPFYRFVSKDMMTDLSFPLKSNTVPIWSVPVYAMLLPLVIFIAIYFRRRDVYDLHHAVLGLLYSVLVTAVLTDSIKNAVGRPRPDFFWRCFPDGKAFYDSLGDVICHGDKSVIREGHKSFPSGHTSWSFAGLGFLSLYLSGKIQAFDGKGHVAKLCIVILPLLVAALVGISRVDDYWHHWQDVFAGGLLGLVVSTFCYLQFFPPPYHTEAWGPYAYFQVLEAARAQAQAAENGVAQGGNGEEEDGGFMGLHLVDNPSMRREDEDVEAGRVPRG

>BnLPP3.3

MMREAQQGTHTVRSHGMTLARTHMHDWIILVLLVILECILLIIHPFYRFVGKDMMTDLNYPLKSNTVPIWSVPVYAMLLPLAIFVFIYFRRRDVYDLHHAVLGLLYSVLVTAVLTDSIKNAVGRPRPDFFWRCFPDGKAVYDSLGDVICHGDKSVIREGHKSFPSGHTSWSFAGLGFLSLYLSGKIRAFDGKGHVAKLCIVILPLLVAALVGISRVDDYWHHWQDVFAGGLLGLVVSTFCYLQFFPPPYRTEAWGPYAYFLVLEAARAQAQAAENEAVQRPPQGDNGEEEDGGFMGLHLVDNPSMRREEADVEAGRVPSRG

>BnLPP3.4

MMREAHQSTHTVRSHGMTLARTHMHDWIILVLLVILECILLIIHPFYRFVGKDMMTDLSYPLKSNTVPIWSVPVYAMLLPLAIFIFIYFRRRDVYDLHHAVLGLLYSVLVTAVLTDSIKNAVGRPRPDFFWRCFPDGKAVYDALGDVICHGDKSVIREGHKSFPSGHTSWSFAGLGFLSLYLSGKIRAFDGKGHVAKLCIVILPLLVAALVGISRVDDYWHHWQDVFAGGLLGIVVSTICYLQFFPPPYRTEAWGPYAYFLVLEAARAQAQAAENEMAQRPPQGDNGEEEDGGFMGLHLVDNPSMRREEADVEAGRVPSRG

>BnLPP4.1

MGKITLGSHTVKSHGWKVAREHLIDWIILVLLGIVIIVLNIMEPFHRYIGPDMLTDLTFPLYQDTIPIWAVPIICILVPICIFTVYYYFGRDVYDLHHAILGIGFSCLVTGVTTDSIKNAVGRPRPNFFHRCFPDGKAKFDSVTKDVVCHGLKKIIKEGYKSFPSGHTSWSFAGLTFLAWYLSGKIKVFDRRGHVAKLCLVFLPILVAVLIGITRVDDYWHHWTDVFAGAIIGIFFASLSYLHFFPYPYDDNGWAPHAYFRMLAESSRRDTTMTRSSSREMLENDVELGSTSMPHHRNRGSTDSE

>BnLPP4.2

MGKITLGSHTVKSHGWKVAREHLIDWIILVLLGIVIIVLNIMEPFHRYIGPDMLTDLTFPLYQDTIPIWAVPIICILVPICIFTVYYYFGRDVYDLHHAILGIGFSCLVTGVTTDSIKNAVGRPRPNFFHRCFPDGKAKFDSVTKDVVCHGLKKIIKEGYKSFPSGHTSWSFAGLTFLAWYLSGKIKVFDRRGHVAKLCLVFLPILVAVLIGITRVDDYWHHWTDVFAGAIIGIFFSSLSYLHFFPYPYDDNGWAPHAYFRMLAESSRRDTTMTRSGSREMLENDVELGSTSMPHHRNRGSTDSE
